# Targeted sequencing of both DNA strands barcoded and captured individually by RNA probes to identify genome-wide ultra-rare mutations

**DOI:** 10.1038/s41598-017-03448-8

**Published:** 2017-06-13

**Authors:** Qing Wang, Xu Wang, Pheobe S. Tang, Grace M. O’leary, Ming Zhang

**Affiliations:** 1 MyOmicsDx Inc., 600 Washington Avenue Suite 100-W, Towson, Maryland 21204 USA; 2 MyOmicsDx (Beijing) Inc., North Yong Chang Road Building # 3-3, Economic-Technological Development Area, DaXing, Beijing, 100176 P.R. China; 3 Clinical Proteomics Technologies Inc., 8101 Sandy Spring Road, Laurel, Maryland 20707 USA

## Abstract

Next Generation Sequencing (NGS) has been widely implemented in biological research and has made a profound impact on patient care. One of the essential NGS applications is to identify disease-causing sequence variants, where high coverage and accuracy are needed. Here, we reported a novel NGS pipeline, termed a **Seq**uencing System of **D**igitalized Barcode **E**ncrypted Single-stranded Library from **E**xtremely Low (quality and quantity) DNA Input with **P**robe-based DNA **E**nrichment by **R**NA probes targeting DNA duplex (***DEEPER-Seq***). This method combines an ultra-sensitive single-stranded library construction with barcoding error correction, termed ***DEEPER-Library***; and a DNA capture approach using RNA probes targeting both DNA strands, termed ***DEEPER-Capture***. ***DEEPER-Seq*** can create NGS libraries from as little as 20 pg DNA with PCR error correcting capabilities, and capture target sequences at an average ratio of 29.2% by targeting both DNA strands simultaneously with an over 98.6% coverage. Our method tags and sequences each of the two strands of a DNA duplex independently and only scores mutations that are found at the same position in both strands, which allows us to identify mutations with allelic fractions down to 0.03% in a whole exome sequencing (WES) study with a background error rate of one artificial error per 4.8 × 10^9^ nucleotides.

## Introduction

NGS is revolutionizing biomedical research and clinical patient care by analyzing billions of DNA base pairs in a high-throughput but relatively low-cost manner^1^. NGS-based detection of single nucleotide variants (SNVs) is one of the pivotal goals that most NGS applications are striving to achieve^2–4^. One typical example would be the NGS-based early diagnostics of human cancers. Due to the heterogeneous nature of human cancers, a successful cancer-causing SNV identification is dependent on the NGS pipelines’ performance of detecting rare mutations at low allelic fractions. NGS-based early cancer diagnostics is of particular interest to many scientists and doctors; however, the most critical but unsolved issue is to differentiate real mutations from artificial sequencing errors. Polymerase chain reaction (PCR) plays an essential role in NGS, but it inevitably creates artificial errors during library preparation, thereby limiting the accuracy of NGS mutation calling^5^. It is extremely hard to distinguish an artificial error from a real SNV when the SNV’s allelic fraction is as low as the frequency of a PCR-based error. Numerous targeted amplification and sequencing methods have been proposed to reduce PCR errors, but all of them can only be applied at several specific short subgenomic regions^3, 6–8^. There is by far no method to discover rare mutations from limited quantities of clinical samples on the scale of whole exome with intrinsic error-correcting capabilities.

There are two main reasons why rare sequence variants are drawing increasing attention in biomedical research. First, in NGS-based basic research, until now, the majority of critical disease-causing variants have already been identified for a large amount of important diseases where a plateau is being reached^9^, and it is now a pivotal task to reveal how those less frequent variants are working together to further fine tune the disease onset and progress; Secondly, in NGS-based clinical applications, researchers and doctors often have to deal with many tough issues coming along with the clinical samples, where the diseased samples can be highly heterogeneous and blended with normal tissues, particularly for tumor samples, thus making the true disease-causing variants present at very low allelic fractions and become rare-mutations. Given the aforementioned limitations and demands, to detect mutations at very low frequency is needed for many NGS clinical applications. However, there are three major problems limiting NGS-based rare mutation detection^10^. First, NGS data will inevitably contain artificial errors (~1% of the bases) that arise during PCR amplifications in sample preparation and sequencing steps, and such errors have to be corrected in order to identify rare SNVs. Secondly, to observe a rare mutation, sufficient depth of coverage is necessary, but is usually not achieved. For example, to detect a mutation with 1% allelic fraction, there must be at least 100× coverage for that particular base to allow the sequence variant to be observed once. However, the depth of coverage is usually limited by the sequencing platforms’ specifications and the quality of sample preparation. Thirdly, the amount of DNA material is usually limited, particularly for a screening test designed to be applied to a large general population, where usually only several milliters of blood are collected from each individual.

Single strand library construction is intrinsically much more sensitive than a standard double-stranded library construction workflow and has been developed to prepare tough and limited DNA materials for NGS analysis^11, 12^. It treats each DNA single strand molecule as a basic unit for library construction. A standard single-stranded library protocol is capable of constructing libraries from as low as 3 × 10^8^ double-stranded DNA molecules (with an average fragment size of 150 bp from 50 pg of DNA)^12^. With a similar un-barcoded single-stranded library protocol, Peng *et al*. constructed a ChIP**-**seq library from as little as 25 pg of DNA^11^. However, none of the reported single-stranded library pipelines incorporated barcoding at the single-stranded DNA level, therefore none of them can perform error-correction.

DNA exists naturally as a double-stranded molecule, where one strand is encoded as a complementary molecule to the other strand. Based on this information redundancy, if the sequences of the two strands can be determined individually, it is possible to correct most if not all PCR and sequencing errors by calling a perfect match between the two complementary DNA strands. Such methods were termed as “duplex sequencing”. Several NGS studies have reported that, through duplex DNA sequencing approach and scoring only the SNVs with matched complementary sequences on both DNA strands, PCR errors and sequencing errors can be removed^10, 13^. The background error rate of such methodology can be estimated as <6.45 × 10^−14^, given the fact that the error rate of Q5® or Q5® Hot Start DNA Polymerase is <4.4 × 10^−7^ as reported by New England BioLabs Inc; therefore, ideally the possibility of having matched artificial SNV errors at the same position on both DNA strands is 1/3 × (4.4 × 10^−7^)^2^ = 6.45 × 10^−14^. This extremely low theoretical error rate makes duplex sequencing an ideal strategy to identify ultra-rare mutations. However, all duplex sequencing methods reported to date are based on the standard double-stranded library construction method which is very vulnerable to DNA damages^12, 13^, therefore they are far less sensitive than single-stranded library construction. In addition, double-stranded barcodes can be added only after the end repairs or other repairs on the original DNA molecules, which made artificial errors arising during these initial repair steps inaccessible.

In contrast to the traditional duplex sequencing, to treat each DNA single strand as an individual molecule is critical for NGS library construction from damaged or very low quantities of DNA materials^12^. Each single-stranded DNA molecule could come from a perfectly intact DNA duplex molecule, or a partially degraded DNA duplex molecule where a fragment of a single strand is missing, or a slightly damaged DNA duplex molecule where a couple of bases are damaged or missing and a few gaps are present, etc. Due to the nature of single-stranded library construction, it is tolerant to all these common damages. Furthermore, to tag each single-stranded DNA molecule with a unique barcode at the very first step before any end repair can abolish any possibility of introducing uncontrollable artificial errors into the DNA sequences during end repair steps. Those improvements can facilitate a highly efficient sequencing of both DNA strands independently from tough samples with extreme error correction capabilities implemented by individual barcoding on each strand. A perfect match between complementary DNA sequences with different barcodes can be called in data analysis to further pinpoint the real mutation.

High efficiency of the barcoded error-correcting system and sequencing on both strands depend on a stringent filter and a large number of unique reads. In addition, to detect rare SNVs, a great depth of coverage for every base is necessary. However, it is usually economically prohibited to identify rare SNVs from the entire human genome, where at least hundreds of folds of coverage are needed for every single base. Instead, exome sequencing or targeted gene panel re-sequencing allows standard NGS platforms to reach a great depth of coverage by limiting the genomic regions to be sequenced. To detect rare mutation from WES study requires a large number of highly enriched DNA molecules captured by probes. However, major commercial target capture platforms can only capture an average of 9% target sequences from the NGS libraries regardless of using single-stranded RNA or single- or double-stranded DNA probes^14–24^. Although NGS results show that the broadness of coverage of those commercial target enrichment platforms can be higher than 95%, the molecules being physically sequenced are only coming from less than 10% of the target sequences in library. Such low enrichment efficiency will inevitably create biases for NGS results or lead to failure of enriching the mutant allele when its allelic fraction is too low. Therefore, an improved target sequence enrichment method needs to be established to highly efficiently and specifically capture the desired subgenomic DNA regions and to provide a molecule library pool large enough for downstream NGS-based rare mutation detection.

In the work described herein, we create a new NGS sequencing pipeline, called “***DEEPER-Seq***” and show that this pipeline offers an improved workflow to conduct error-correction enabled NGS research with very limited DNA input by constructing a single-stranded DNA library using uniquely barcoded DNA single-stranded molecules, followed by highly efficient enrichment of both complementary strands of DNA library molecules simultaneously through using a massive amount of RNA hybridizing probes. The barcode on each DNA single strand allows the error-correcting capabilities by consolidating DNA sequences bearing the same unique barcode. Our ***DEEPER-Library*** is by far the only library construction method that adds barcode to each individual DNA single strand through direct ligation, but not by PCR, before any repair process, thus maximizing the performance of error correction. And our ***DEEPER-Capture*** method is the only method that captures both DNA strands simultaneously through complementary RNA probes and repeatedly achieves an over 3-fold increase in the capture efficiency compared to the efficiencies observed from the best performance of all major commercial target enrichment platforms^14–18^. The ***DEEPER-Seq*** system is a revolutionary combination of both extreme sensitivity and ultimate specificity for the most demanding tasks in an NGS study, where it allows rare SNVs to be detected at the whole exome scale from very low amount of highly heterogeneous materials.

## Material and Methods

### Tumor and normal tissue sample

The paired tumor and normal tissue samples from a pancreatic cancer patient of Asian race were obtained in accordance with guidelines and regulations from Tianjin Medical University Cancer Institute & Hospital, P.R. China after Institutional Review Board (IRB) approval at Tianjin Medical University, and under full compliance with HIPAA guidelines. An informed consent for conducting this study was obtained from the patient. The tumor tissue sample has an estimated neoplastic content of 43.4%.

### DEEPER-Library preparation

**Figure 1:**
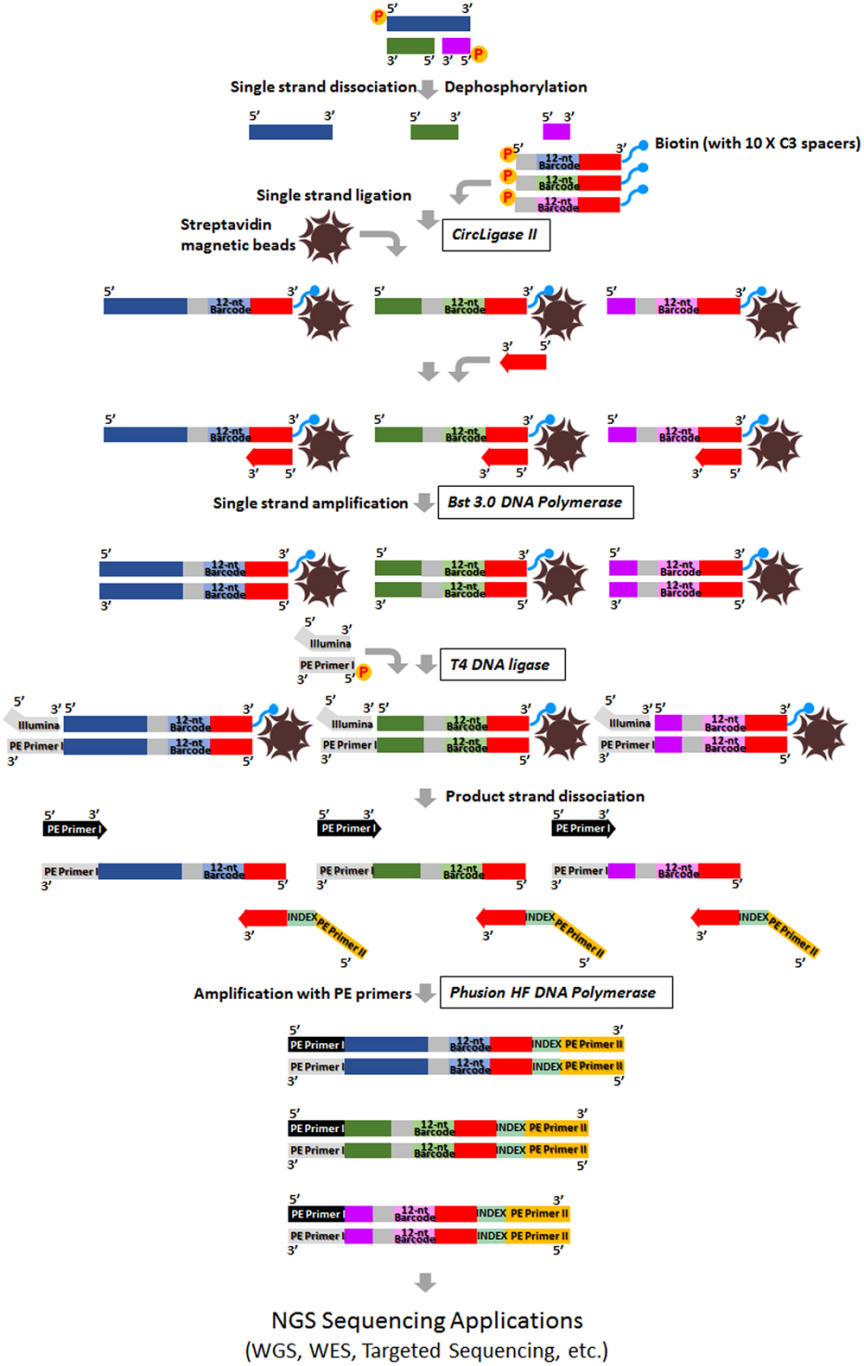
Overview of ***DEEPER-Library*** construction. A double-stranded DNA molecule (bearing a damaged strand or not) is heated to dissociate complementary DNA single strands. A barcoded (12nt) single-stranded adapter is appended to the 3′ end of a single-stranded DNA molecule and the entire molecule is immobilized on a streptavidin-bead. A PCR primer complementary to the 3′ sequence on every adapter is added as a primer to synthesize the complementary sequences of the initial single-stranded DNA molecule and the barcode. Illumina PE sequencing adapter is appended to the 3′ end of the newly synthesized complementary single-stranded DNA. PE primer I and a joined primer of single - stranded adapter – index - PE primer II are used to amplify the DNA fragments in the library. After amplification, the library is ready for direct NGS sequencing or subgenomic capture for targeted sequencing.

Genomic DNA from patient normal and tumor fresh frozen tissues were extracted using DNeasy Blood & Tissue Kit (Qiagen) and sheared into 150 bp fragments with Diagenode’s Bioruptor at a program of 7 cycles of 30 seconds ON/90 seconds OFF using 0.65 ml Bioruptor® Microtubes. ***DEEPER-Library*** preparation starts from a complete dissociation of DNA duplex to form single-stranded DNA and tagging the 3′ end of each DNA single strand individually with a unique digital barcode. Barcoded single-stranded adapters were synthesized from Integrated DNA Technologies with the sequence format as 5′-Phosphorylation Modification-CCCAA-NNNNNNNNNNNN-CCTCAGCAAG-XXXXXXXXXX-TEG-biotin, where each “X” indicates a unit of C3 spacer. Pre-dephosphorylated fragmented DNA samples were mixed with barcoded single-stranded adapter (final concentration 0.15uM), 20% PEG-8000, 100U CircLigase II, and incubated at 60 °C for 1 hour. After immobilizing the ligation product on Streptavidin-coupled Dynabeads (ThermoFisher Scientific), each barcoded single-stranded DNA molecule is subject to an individual single-cycled PCR reaction to form its complementary strand. A DNA primer complimentary to the single-stranded adapter was annealed and extended using Bst 3.0 polymerase at 50 °C for 30 minutes. Blunt-end repair using T4 DNA polymerase was performed at 25 °C for 15 minutes. A double-stranded adapter was then ligated to the 5′ end of the DNA duplex using T4 DNA ligase with an incubation at 16 °C for 1 hour. The library is eluted from the beads by an incubation at 95 °C for 1 minute. High fidelity PCR amplification is performed to amplify the DNA sequence as well as the unique barcode. Adapter sequences are designed to be compatible with Illumina sequencing platforms. ***DEEPER-Library*** construction procedure can be outlined in Fig. 1.

### *DEEPER-Capture* RNA probe synthesis

**Figure 2:**
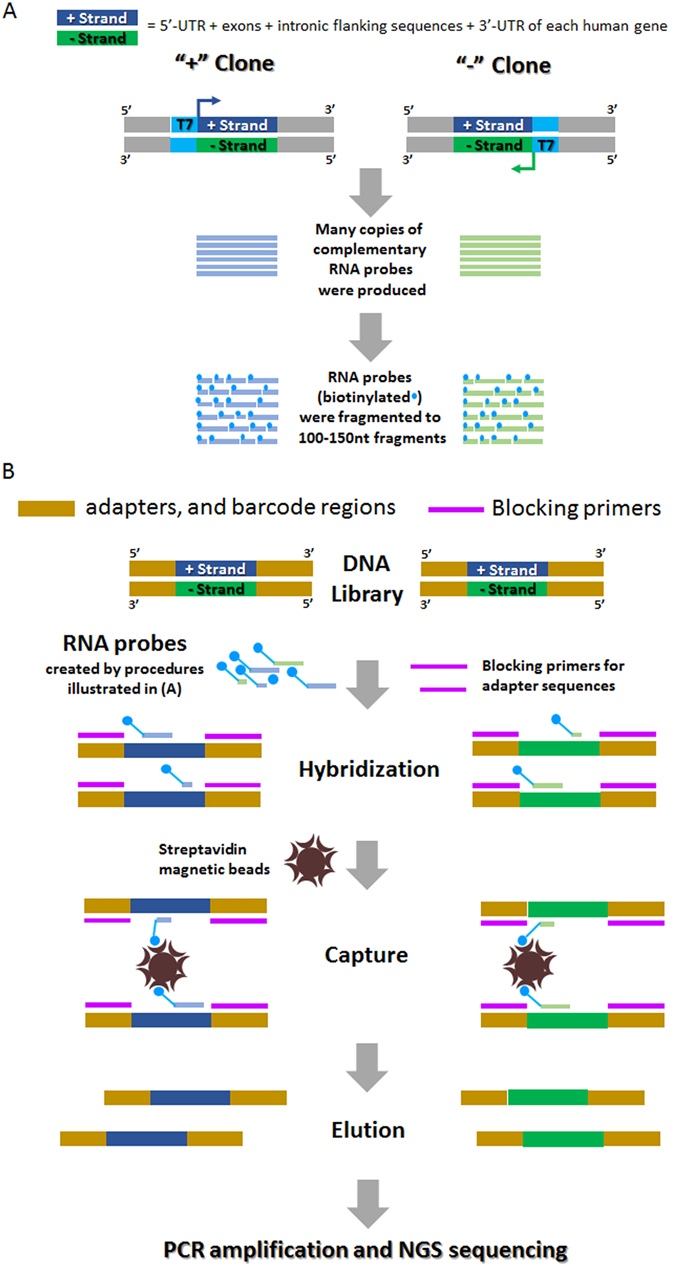
Overview of ***DEEPER-Capture*** procedure. (**A**) Overview of RNA probe production. Target DNA sequences are cloned into pcDNA 6.2 vector. Two clones for each target sequence are constructed, with a T7 promoter inserted upstream of the target sequence on either strand. For each DNA target sequence, in the “+” clone, T7 promoter is inserted at the 5′ end of the plus strand of the target sequence; and in the “−” clone, T7 promoter is inserted at the 5′ end of the minus strand of the target sequence. “+” clone and “−” clone for each target sequence are separated into 2 pools and are subject to *in vitro* transcription to produce biotin labeled RNA probes individually. 2 pools of RNA probes are then sheared into 100-150nt RNA fragments by ultrasonication. (**B**) Overview of ***DEEPER-Capture*** workflow. A hybridization mixture is prepared by first heated dissociation of double-stranded DNA molecules in the library, blocking primers and two pools of RNA probes are added into the system sequentially in a heated environment. After hybridization, streptavidin beads are used to isolate RNA probes and their hybridized DNA targets from the system. Target DNA sequences are then eluted from the beads and subject to PCR amplification and NGS sequencing.

To obtain RNA probes complementary to both strands of target subgenomic regions, particularly the exome regions, we cloned the entire exome sequences for every human gene by sequence synthesis and molecular cloning based on Hg19 reference human genome sequences. In brief, exome sequences in 32,524 CCDS IDs containing −50bp and +50 bp intronic sequences were cloned into pcDNA 6.2 vector. For extremely large human genes, e.g. DMD, PTPRD, CNTNAP2, etc., their related target sequences were separated and subcloned into multiple vectors. The total DNA sequences we used to generate RNA probes cover a 72.6 Mb genome region, where all the exomes with their −50 bp and +50 bp flanking intronic sequences, as well as 5′ and 3′ UTRs for each gene were included. Two clones for each target sequence were constructed, where a T7 promoter was inserted at the 5′ end of the plus strand in the “+” clone and at the 5′ end of the negative strand in the “−” clone (Fig. 2A). Two pools of clones were established for any given number of genes following the rule that the two clones for the same DNA sequence are separated into two systems, where one system (Fig. 2A, “+” Clone) produced the RNA probes targeting the plus strand of the DNA target, and the other system (Fig. 2A, “−” Clone) produced the RNA probes targeting the minus strand of the DNA target through *in vitro* transcription. ATP, CTP, GTP, UTP, and Biotin-16/11-UTP were added in each transcription system at the concentration of 1 mM, 1 mM, 1 mM, 0.7 mM and 0.3 mM. RNA products were further sheared into 100–150nt fragments with a Covaris S220 focused-ultrasonicator (Covaris). The fragmented RNA probes are ready for ***DEEPER-Capture*** applications. The two RNA probe libraries for each target DNA sequence were created separately and were never mixed until the actual ***DEEPER-Capture*** procedure was carried out. In this study, we created ***DEEPER-Capture*** probes targeting whole exome sequences (including −50bp and +50 bp flanking intronic sequences and 5′/3′ UTRs) for all human genes and a cancer-related 298-gene panel.

#### DEEPER-Capture

***DEEPER-Capture*** was performed to capture the whole exome of human genome following a ***DEEPER-Library*** construction or a standard NGS library construction. In ***DEEPER-Capture***, both DNA strands of the target regions are captured by a pair of complementary RNA probes where each DNA strand is targeted by its complementary RNA probe, individually. A hybridization mixture was prepared containing 500 ng ***DEEPER-Library*** DNA, 2 ug of RNA probes (1 ug from “+” clone transcripts and 1 ug from “−” clone transcripts, targeting Hg19 human exomes including −50 bp and +50 bp flanking intronic sequences, as well as 5′ and 3′ UTRs as described before), 7 ul Human Cot-1 DNA (ThermoFisher Scientific), 3 ul Herring Sperm DNA Solution (ThermoFisher Scientific), 10 µl blocking Oligos (1 nmol/ul each) with following sequences:

Blocking Oligo 1:

5′-AAGAGCGTCGTGTAGGGAAAGAGTGTAGATCTCGGTGGTCGCCGTATCATT-3′ Inverted dT

Blocking Oligo 2:

5′-CCTCAGCAAGAGCACACGTCTGAACTCCAGTCAC-7 mer Index-ATCTCGTATGCCGTCTTCTGCTTG-3′ Inverted dT

The hybridization mixture is heated for 5 minutes at 95 °C, then held at 67.5 °C. 25 ul pre-warmed (67.5 °C) 2.8 X hybridization buffer (14 X SSPE, 14 X Denhardt’s, 14 mM EDTA, 0.28% SDS) was added. The mixture was slowly pipetted up and down 8 to 10 times. The hybridization mixture was incubated for 24 hours at 67.5 °C with a heated lid.

After hybridization, 50 µl Dynal MyOne Streptavidin C1 magnetic beads (ThermoFisher Scientific) were washed three times by adding 200 µl of binding buffer (1 M NaCl, 10 mM Tris-HCl, pH 7.5, and 1 mM EDTA), and re-suspended in 200 µl Binding buffer. The hybridization mixture was added to the bead solution gently and was subsequently incubated on a thermomixer at 850 rpm for 30 minutes at room temperature. To wash the beads, supernatant was removed from beads on a Dynal magnetic separator and the beads were re-suspended in 500 µl Wash Buffer A (1X SSC/0.1% SDS), and incubated for 15 minutes at room temperature. Beads were then washed three times, each with 500 µl pre-warmed Wash Buffer B (0.1XSSC and 0.1% SDS) after incubation at 65 °C for 10 minutes. To elute captured DNA, beads were re-suspended in 50 µl 0.1 M NaOH at RT for 10 minutes. The supernatant was transferred into a new 1.5 ml microcentrifuge tube after magnetic separation, and mixed with 50 µl Neutralizing Buffer (1 M Tris-HCl, pH 7.5). DNA was purified with a Qiagen MinElute column and eluted in 17 µl of 70 °C EB buffer to obtain 15 µl of captured DNA library. The captured DNA library was amplified by Phusion Hot Start polymerase (New England Biolabs) using Illumina PE primer 1 and 2. The PCR program used was: 98 °C for 30 seconds; 6~10 (depending on capture yield) cycles of 98 °C for 10 seconds, 65 °C for 30 seconds, 72 °C for 30 seconds; and a final incubation at 72 °C for 5 minutes. The PCR product was purified using GeneJET PCR Purification kit (ThermoFisher Scientific).

### Real-time PCR assay

Real-time PCR assays with SYBR green detection was carried out using an ABI PRISM 7500 Sequence Detection System (Applied Biosystems). More details are provided in the Supplementary Methods.

### Whole exome sequencing

Whole exome sequencing was performed on an Illumina HiSeq 2500 platform according to manufacturer’s manual. Total number of on-target reads from randomly chosen 5 million to 50 million reads were calculated. After trimming and barcoded super read grouping, SNVs were called with GATK (version 3.6) in a default mode as recommended by the GATK documentation with reference genome of Hg19^25^. In brief, for every sample (tumor or normal DNA), sequencing result was preprocessed by mapping to reference genome with BWA (version 0.7.10), and duplicates were marked with Picard (version 2.0.1). Base Recalibration was performed to generate the reads ready for SNV analysis. For individually processed T/N pair reads, Indel Realignment was performed to generate pairwise-processed T/N pair reads. HaplotypeCaller was used for raw SNV calling. Output from variant calling was directly used for SNV detection by MuTect (version 1)^26^. Mutations were filtered through a 4-step approach introduced in the section “Mutation and ultra-rare mutation detection”. Low-quality variant with a Phred score <30.0 was abandoned. Paired SNVs from complementary reads bearing different barcodes were identified as true mutations and subject to further validation through Sanger sequencing. The data yields after each step of data analysis for ***DEEPER-Seq*** were shown in Table 1. SNVs identified and Sanger sequencing validation results were provided in Supplementary Table S2.

**Table 1:** Data yield from *DEEPER-Seq* WES sequencing.

	Normal Tissue	Tumor Tissue
Initial mapped reads	30.7 billion	25.7 billion
Average raw coverage	240.5×	173.9×
Unique read family (URF)	625 million	577 million
Super read duplexes	272 million	231 million
Initial mapped reads per super read family	49.1	44.5
Initial mapped reads per super read duplex	112.9	111.3
Super reads per super read duplex	2.3	2.5

Data yield from ***DEEPER-Seq*** WES sequencing. *Initial mapped reads* represent raw reads that contain the 12nt barcode and mapped to the reference genome. *Unique read family* represents the number of URF. Each URF has a unique barcode and its sequence is obtained by consolidating read sequences arise from the same DNA molecule by PCR amplification. PCR errors are removed by requesting a sequence uniformity for over 95% of the reads within a URF. *Super read duplexes* represent the number of DNA duplex whose two strands are coming from two super reads.

### Mutation and ultra-rare mutation detection

The significantly increased number of unique reads obtained through ***DEEPER-Seq*** approach enabled us to apply our stringent filters with the following 4-step procedure.

Step 1) group reads with the same barcode that are representing PCR duplicates of an original barcoded single-stranded DNA molecule, and call it a unique read family (URF);

Step 2) combine reads within each URF obtained from Step 1) by requesting >95% sequence identity among the reads;

Step 3) extract the unique DNA sequence and the barcode sequence for each URF, and call it a “super read”

Step 4) for all the super reads identified in Step 3), find their paired complementary super reads, and only score sequence variants with matched complementary sequences from paired super reads. To accommodate damaged DNA molecules in the sample, complementary super reads may not be at the same length (Fig. 1).

To evaluate the performance of ***DEEPER-Seq*** in detecting low frequency (ultra-rare) mutations, we sequentially diluted 100 ng tumor DNA sample by 10, 100, 1,000 and 10,000 folds, and spiked each of them into the same amount (100 ng) of genomic DNA extracted from the paired normal tissue of the aforementioned cancer patient. This design can simulate early stages of cancer occurrence, and represent the major obstacles in early cancer diagnostics using NGS, which is the very low allelic fractions of tumor specific mutations in the sample.

### Build a highly accurate reference exome for ultra-rare mutation identification

An updated highly accurate reference exome was built for the patient’s normal genome by repeating the ***DEEPER-Seq*** WES experiments for 6 times. Conceptual and practical procedures related to creating such a high-accuracy reference exome are discussed in detail in Supplementary Methods.

## Results

### Overview

Our approach, called the “***DEEPER-Seq***”, involves two basic steps (Figs 1 and 2B). The first is the barcoded single-stranded library (***DEEPER-Library***) construction. The second is the enrichment of both DNA strands from the same DNA molecule (DNA duplex) through complementary RNA probes (***DEEPER-Capture***). Both steps can be performed together or independently adopted and incorporated into standard NGS pipelines.

#### DEEPER-Library

**Figure 3:**
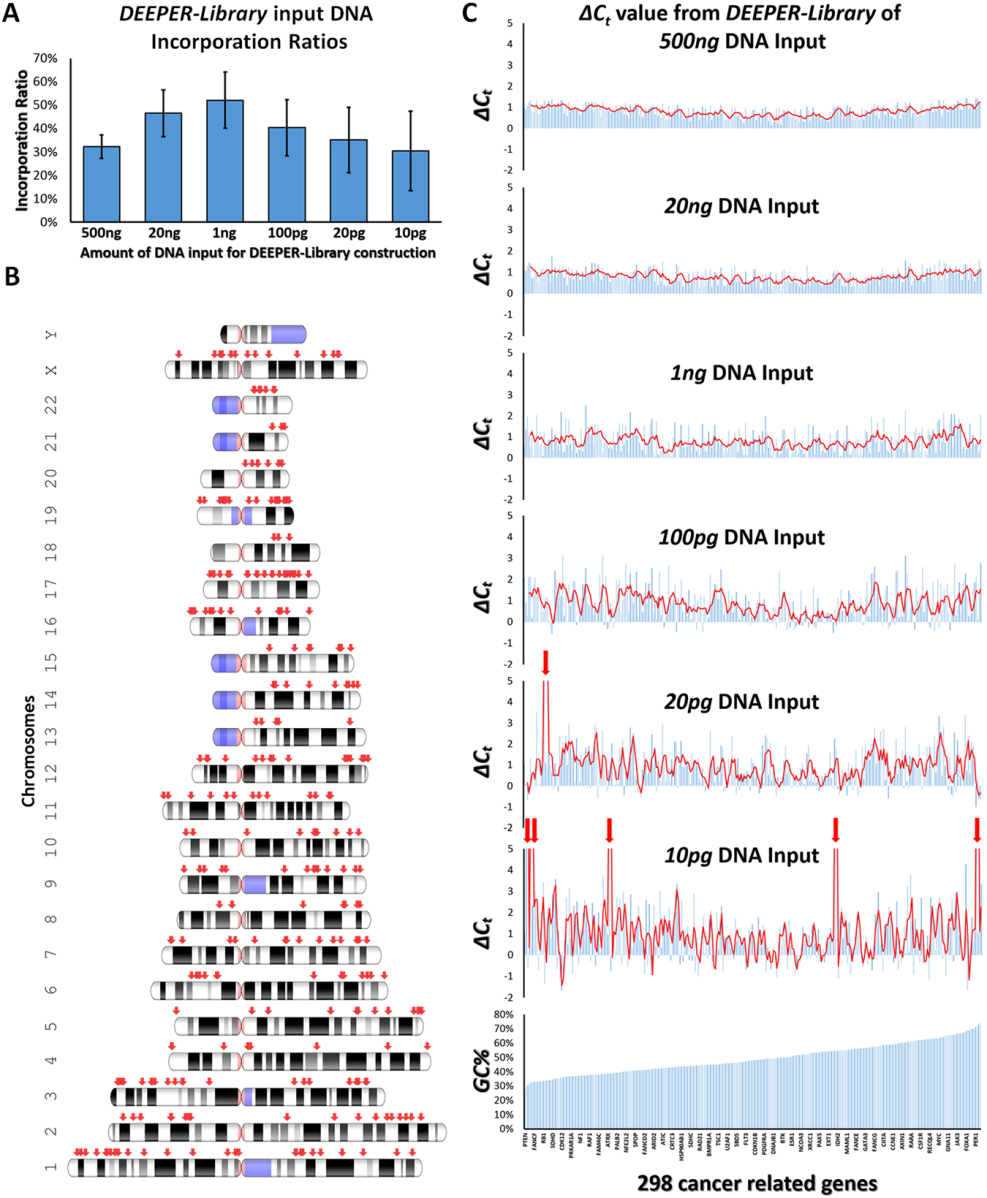
Performance evaluation of ***DEEPER-Library***. (**A**) Incorporation ratios of single stranded DNA in ***DEEPER-Library*** pipeline. The fractions of DNA molecules that were incorporated into ***DEEPER-Library*** construction from different amounts of starting DNA (500 ng, 20 ng, 1 ng, 100 pg, 20 pg and 10 pg genomic DNA) were plotted. Ratios were measured by Qubit® ssDNA Assay Kit on a Qubit unit (ThermoFisher Scientific). (**B**) Genomic locations of 298 cancer related genes on human chromosomes (indicated by red arrows). (**C**) Six *ΔC*_*t*_ values for each of the 298 genes were calculated by real-time PCR assays detecting gene abundance difference between 500 ng original genomic DNA input and 500 ng ***DEEPER-Library*** final products created from six different amounts (500 ng, 20 ng, 1 ng, 100 pg, 20 pg and 10 pg) of input genomic DNA. The 298 genes were ranked by the GC% of their amplicons and gene names were shown on the x-axis from low GC% (left) to high GC% (right).

***DEEPER-Library*** is prepared by a barcoded single strand library construction method. To assess the performance of ***DEEPER-Library*** method in creating valid NGS libraries from limited amounts of DNA materials, we constructed 6 ***DEEPER-Libraries*** from sequentially diluted genomic DNA (500 ng, 20 ng, 1 ng, 100 pg, 20 pg and 10 pg) extracted from the normal pancreas tissue of a cancer patient. The first step of ***DEEPER-Library*** construction is to ligate barcoded single-stranded adapters to single strand DNA molecules, and this step is critical, since it provides the initial pool of DNA molecules for all downstream procedures. The average ligation efficiency for this step measured for 6 libraries were 32.3%, 46.5%, 52.1%, 40.3%, 35.1% and 30.5% (Fig. 3A). These values indicated the incorporation ratios of different amounts of genomic DNA molecules into the ***DEEPER-Library*** workflow. This ratio is essential for successful NGS applications with very limited starting materials and very heterogeneous samples. The ligation proved very efficient that it utilized over 50% of 1 ng genomic DNA molecules, and this ratio remained above 30% with as low as 10 pg genomic DNA input. We performed six ***DEEPER-Library*** constructions and 500 ng library products from each of the 6 libraries were used for further performance evaluation.

298 human cancer related genes located on chromosome 1 through 22 and chromosome X (Fig. 3B) were selected as genome landmarks to indicate the broadness and depth of coverage of ***DEEPER-Library*** as well as the enrichment efficiency and evenness of subgenomic regions by ***DEEPER-Capture***, measured by real-time PCR assays. Gene-specific primer pairs were designed and used to amplify the 298-gene panel (Supplementary Table S1). Seven real-time PCR reactions, with three replicates for each reaction, were performed for each gene using 500 ng genomic DNA and 500 ng library product from each of the six ***DEEPER-Libraries*** created from different amounts of input DNA. After taking the average of triplicates, six *ΔC*_*t*_ values between initial DNA input and six library products were calculated for each gene, and were subsequently plotted to compare across 298 genes for their abundances before and after the ***DEEPER-Library*** constructions with different amounts of starting materials (Fig. 3C). Amplification efficiencies for 298 target amplicons were established and listed in Supplementary Table S1 with an average value of 1.88. The average size of sheared DNA single strands is 150 bp, and the total length of adapter sequences added to each DNA single strand sequence during library preparation is 135nt (Fig. 1). Therefore, the distribution of *ΔC*_*t*_ between 500 ng final ***DEEPER-Library*** products and 500 ng initial DNA input fragments should be presumably centered at log_1.88_[(135 + 150)/150] = 1.017, which was in consistency with the data we observed (Fig. 3C). We successfully detected all target genes in 500 ng original genomic DNA input, and in four (500 ng, 20 ng, 1 ng and 100 pg DNA inputs) out of six ***DEEPER-Libraries***. Only one and five genes were not detected from the libraries constructed with 20 pg or 10 pg DNA, respectively (Fig. 3C). There is no significant GC% dependent abundance bias observed from *ΔC*_*t*_ values for all genes. More importantly, despite the different amounts of DNA materials to start with, ***DEEPER-Library*** evenly amplified the entire human genome landmarked by the panel of 298 genes. We re-designed PCR primers targeting a different genomic region for each of the six genes that were not detected in the two most diluted DNA samples (20 pg and 10 pg), and re-performed the same set of seven real-time PCR assays for each gene. Positive results were observed using new primers (Supplementary Table S1).

Our results demonstrate that ***DEEPER-Library*** construction method is able to create DNA library from very low DNA material amount (10~20 pg) and generate NGS feasible library products (>1 ug) with high broadness of coverage. ***DEEPER-Library*** has no obvious GC content bias and library molecules are evenly amplified to represent the abundances of original input DNA’s genome sequences. These results also indicate that it becomes less efficient to amplify certain subgenomic regions when the DNA input amount is extremely limited, i.e. around or lower than 20 pg. To construct DNA libraries with extremely low amount of DNA, a whole genome pre-amplification may be necessary. However, such procedure may generate artificial errors before the initial barcoding step in ***DEEPER-Library***, and can hinder its rare mutation detectability. Therefore, we stopped trying to further test any lower amount of DNA materials for library construction, and noted the minimal input limit for a successful ***DEEPER-Library*** construction as 20 pg DNA. This amount (20 pg) contains the total DNA materials from less than 3 human somatic cells. The vast majority of biological samples will be more than enough to offer such abundance level of DNA materials, and ***DEEPER-Library*** has demonstrated an excellent performance in creating NGS libraries with such low amount of DNA.

#### DEEPER-Capture

**Figure 4:**
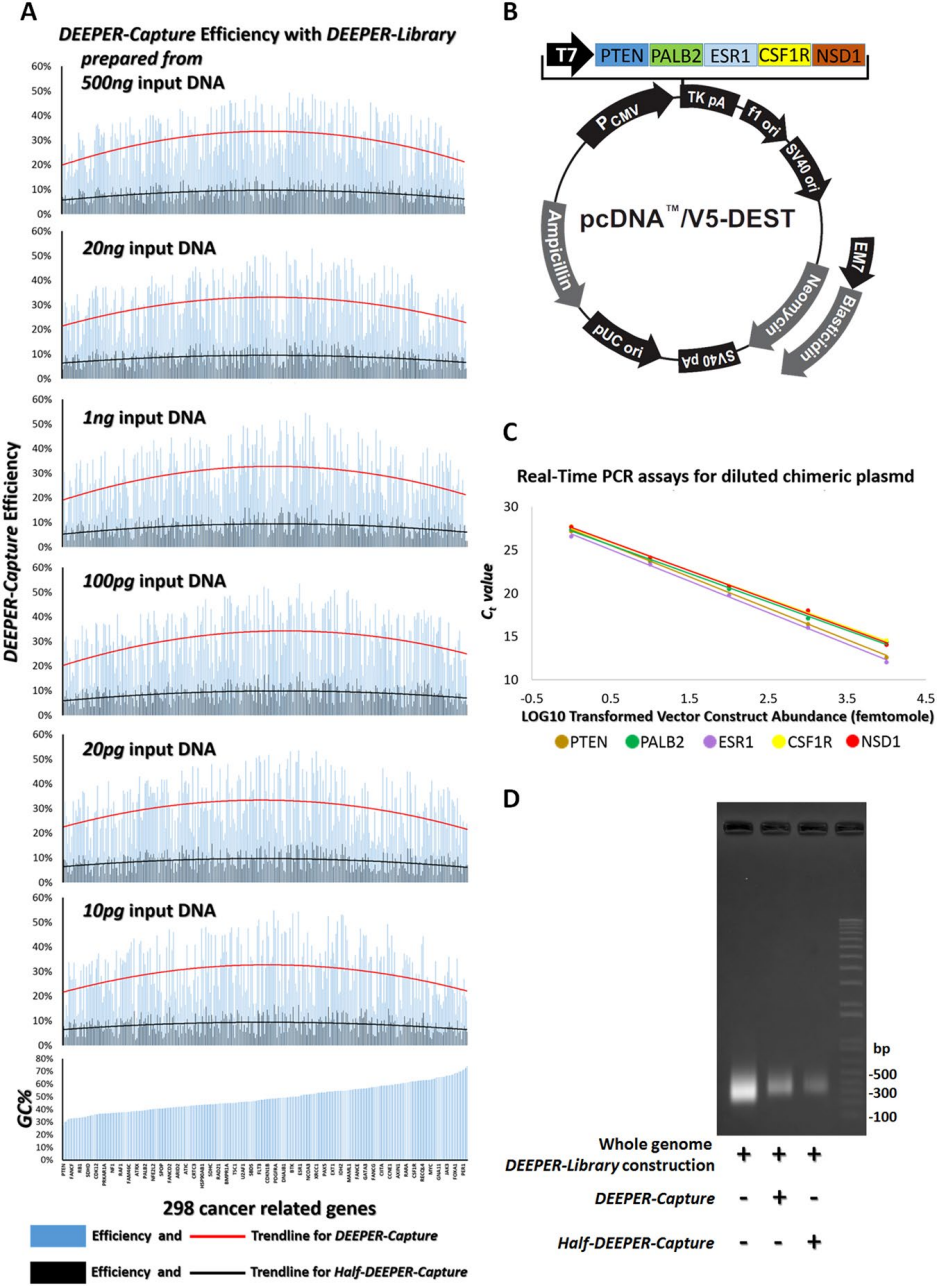
Performance evaluation of ***DEEPER-Capture***. (**A**) For each of the six ***DEEPER-Libraries*** derived from different amounts (500 ng, 20 ng, 1 ng, 100 pg, 20 pg and 10 pg) of input genomic DNA, enrichment efficiencies of 298 cancer related genes were calculated. Recovery ratios of ***DEEPER-Capture*** and ***Half-DEEPER-Capture*** for all 298 genes in six libraries were quantified by real-time PCR assays detecting each gene’s abundance in the libraries before and after ***DEEPER-Capture*** or ***Half-DEEPER-Capture***. (**B**) An insert sequence composed of amplicon regions of five genes whose GC contents fall into a broad range (27.3% to 74.1%) was cloned into a pcDNA vector. (**C**) Real-time PCR analysis of sequential dilutions of the plasmid. 1, 10, 100, 1,000 and 10,000 femtomoles of the plasmids were added as templates for the assays. *C*_*t*_ value for each gene observed from different plasmid template amount was plotted, and trend lines were shown. No significant GC-dependent amplification bias was observed for real-time PCR assays. (**D**) A whole genome ***DEEPER-Library***, and whole exome ***DEEPER-Libraries*** captured by ***Half-DEEPER-Capture*** and ***DEEPER-Capture*** were analyzed on an agarose gel.

***DEEPER-Capture*** is a method to enrich targeted subgenomic sequences by RNA probe-based capture of both strands from the same DNA molecule, simultaneously. To assess the capture efficacy, we created ***DEEPER-Capture*** probes for exonic regions of the 298-gene panel adopted in this study. Real-time PCR assays were performed to detect and quantify the subgenomic regions of this gene panel in ***DEEPER-Libraries*** before and after ***DEEPER-Capture*** enrichment. No re-amplification of the library was performed after the capture to ensure that the amounts of DNA molecules obtained from ***DEEPER-Capture*** enrichment represent the captured yields for each gene. The same set of six ***DEEPER-Libraries*** created from a sequentially diluted DNA input were adopted again. Our results indicate that each gene’s capture ratio is in consistency for all six libraries (Fig. 4A). Such findings demonstrate that ***DEEPER-Capture*** efficiency for each gene is not dependent on the initial DNA input amount for the library construction, which shows again the highly efficient amplification of ***DEEPER-Library*** construction. However, the capture ratios of different genes did vary to a significant extent (10.4% to 49.8%). Further investigation showed that, ***DEEPER-Capture*** ratios for different genes were loosely correlated to the GC content of their amplicons (Fig. 4A). A very weak correlation (average R^2^ = 0.12) between amplicon GC contents and their ***DEEPER-Capture*** efficiencies were shown (Fig. 4A).

***DEEPER-Library***, ***DEEPER-Capture*** or the Real-time PCR assays are all potentially responsible for this GC content associated enrichment bias. Our previous results have demonstrated that ***DEEPER-Library*** is not significantly biased by GC content (Fig. 3C). Next, real-time PCR was investigated to check its potential GC content bias. We chose 5 genes, PTEN, PALB2, ESR1, CSF1R, and NSD1, with distinct GC% in their amplicon sequences at 27.3%, 39.3%, 50.5%, 62%, and 74.1%, respectively (Supplementary Table S1). A plasmid (pcDNA 6.2 vector) containing a DNA insert composed of all five genes’ amplicon regions separated by a 100 bp flanking sequence is cloned (Fig. 4B) and sequentially diluted to simulate variable amounts of the gene fragments after capture. All 5 gene fragments were cloned in the same plasmid to ensure the equal abundance between different genes in every diluted sample. Real-time PCR was performed to detect the copy number of plasmids by detecting each of the 5 genes in a series of diluted plasmid samples. As shown in Fig. 4C, there is no obvious GC-content-associated bias observed from the real-time PCR amplifications of all 5 genes using our primers. The 298 pairs of primers adopted in real-time PCR assays were designed with their *T*_*m*_ values all falling into a narrow range of 57 °C to 61 °C. This restriction of *T*_*m*_ and short amplicon sequences with similar lengths (~around 150 bp) helped ensure the uniformity of real-time PCR assays for all genes (Supplementary Table S1). Therefore, the only possible step, where the GC content related capture ratio bias is created, should be the ***DEEPER-Capture*** itself. Enrichment bias for hybridization-based subgenomic capture has been reported to be owing to GC content^16^. ***DEEPER-Seq*** based whole exome capture NGS studies were conducted to further assess the impact of GC content to the ***DEEPER-Capture*** efficiency of target subgenomic regions.

It is important to note that in ***DEEPER-Capture***, complementary RNA probes were used to capture both DNA strands of the target regions, and we tried to assess if there is any capture efficiency difference between using only one set of RNA probes to capture only one strand of target DNA (termed “***Half-DEEPER-Capture***”) and using two sets of RNA probes to capture both strands of target DNA simultaneously (***DEEPER-Capture***). ***DEEPER-Capture*** and ***Half-DEEPER-Capture*** were performed in parallel with two equal aliquots (500 ng) of DNA libraries, where each library was created from the same 20 ng genomic DNA. A whole genome ***DEEPER-library***, capture yields from ***Half-DEEPER-Capture*** or ***DEEPER-Capture*** were analyzed on an agarose gel (Fig. 4D). Real-time PCR assays for the 298-gene panel were performed to evaluate the capture ratios of ***Half-DEEPER-Capture*** and ***DEEPER-Capture*** for all the genes (Fig. 4A). The average capture ratio for ***DEEPER-Capture*** is 29.2% across all genes in different libraries, much higher than the ratios observed from ***Half-DEEPER-Capture*** approach (~8.5% on average). Our results have demonstrated that to capture target DNA sequences by complementary RNA probes through hybridizing to both strands of the DNA duplex molecule simultaneously achieved an over 3-fold increase in capture efficiency compared to capturing a DNA single strand alone through RNA probes. Possible reasons for such performance were discussed in *Supplementary Discussion*.

### Whole Exome Sequencing by *DEEPER-Seq*

To evaluate the performance of ***DEEPER-Seq*** in NGS, we performed WES using our method and compared the data to what obtained through standard NGS library preparation with a standard exome enrichment procedure. All libraries were constructed with 100 ng genomic DNA derived from the normal tissue of the cancer patient and 3 technical replicates were performed for each sample. All NGS runs were carried out on the same Illumina HiSeq 2500 platform with the same technical specifications of the runs. As shown in Supplementary Figure S1A, an average of 188 million reads were obtained from ***DEEPER-Seq*** WES, where 98.3% were aligned to human genome, and the total read counts were significantly more (1.6 folds) than that from the standard sequencing pipeline. The higher numbers of reads for ***DEEPER-Seq*** libraries presumably came from the ultra-sensitive single-stranded DNA library construction, and the much more efficient enrichment by capturing both DNA strands (including DNA molecules that have damages ranging from minor single strand breaks to major damages on both strands), simultaneously.

All NGS data were analyzed on the same software pipeline with the same settings. Raw reads were filtered to remove duplicates, multiple mappers, improper pairs, and off-target reads. On average 75.4% reads were retained after filtering (Supplementary Figure S1A). For the reads that were removed, 71.8% were off-target reads, which were mapped to the human genome but outside of the target regions; 21.6% were PCR duplicates; and the remaining reads were mapped to multiple sites of the genome or not mapped at all (Supplementary Figure S1B). Because of the excessive amount of RNA probes used in ***DEEPER-Capture***, off-target enrichment was a concern when we designed the pipeline. However, off-target reads added up to 71.8% of the 22.9% total reads that were removed, therefore only 16.4% of the total reads were off-target reads (Supplementary Figure S1B). ***DEEPER-Capture***’s off-target ratio was comparable to other capturing methods, but achieved a significant increase in the yields for target molecules compared to the ***Half-DEEPER-Capture*** and other similar approaches (Fig. 4A). No statistically significant difference was observed in all the specifications measured for the three technical replicates in this experiment, which indicates that ***DEEPER-Seq*** pipeline is technically highly reproducible (Supplementary Figure S1A,B).

Next, we evaluated the correlation between coverage efficiency and sequencing depth in ***DEEPER-Seq***. We utilized the method proposed by Clark *et al*.^16^, and randomly selected filtered reads in 5 million read increments from 5 million to 50 million. The fractions of the retained on-target reads covering the depths of at least 10×, 20×, 50×, and 100× were plotted using randomly selected 5 to 50 million reads (Supplementary Figure S1C). 20 million reads could cover close to 90% of the target bases with no less than 10× depth. With 50 million reads, over 90% target bases were covered by at least 20×. The efficiency of coverage is not only dependent on the efficiency of ***DEEPER-Seq***, but also dependent on the length of the sheared molecules that were initially incorporated into the pipeline. For the current study, the average length of sheared DNA molecule is 150 bp.

Lower coverage in sequencing regions with extreme GC contents has been reported^27, 28^. In a WES experiment, this bias is shown to be derived from the PCR amplifications during library preparation^29^, and the hybridization between probes and target regions during capture^30^. Our real-time PCR results for the 298-gene panel indicated that enrichment efficiency of the ***DEEPER-Seq*** approach is not significantly biased by GC content (Fig. 3C), and ***DEEPER-Capture*** efficiency for each gene in a ***DEEPER-Library*** was only loosely correlated to the GC content of the target gene’s sequence (Fig. 4A).

To assess the impact of GC content on ***DEEPER-Seq*** WES result, we plotted normalized mean read depth against GC content. There is a correlation between GC content and read depth in the ***DEEPER-Seq*** WES experiment (Supplementary Figure S2A), and this bias is reduced in a WGS study using the same ***DEEPER-Library*** without ***DEEPER-Capture*** (Supplementary Figure S2B). In ***DEEPER-Seq***, the mean read depth ratio of GC50%/GC20% = 1.55, and is significantly lower than the ratio of 2.0 reported by numerous studies^31, 32^, which demonstrates a lower GC bias in our method. The improved evenness of enrichment over other pipelines is presumably due to the significantly increased yields of target sequences by ***DEEPER-Capture*** that is capturing both DNA strands together with strong RNA probes.

### Detection of SNVs by *DEEPER-Seq* WES

**Figure 5:**
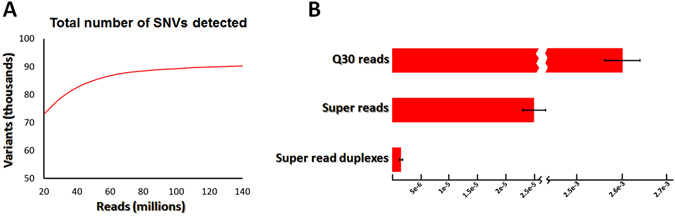
SNV-calling trends and statistics of ***DEEPER-Seq*** WES study. (**A**) Total number of SNVs detected at increasing read count thresholds. Sensitivity increases at higher read counts but quickly reaches a plateau with more than 80 million reads. (**B**) Average SNV frequencies of normal tissue DNA measured by three approaches: a standard NGS approach where barcodes were directly trimmed off, a super read based approach by ***DEEPER-Seq*** without matching variants from both DNA strands (without the last step of the 4-step procedure), and a super read approach by ***DEEPER-Seq*** matching the SNV on both strands (all steps in the 4-step procedure were performed).

One of the most important goals of exome sequencing is to identify sequence variants that are disease-causing or of clinical significance. To evaluate the sensitivity and specificity of sequence variant identification by ***DEEPER-Seq***, we conducted a WES study with 100 ng genomic DNA from a pair of normal and tumor tissue samples obtained from the same cancer patient. The same SNV calling pipeline was used for all data analysis in this study. Briefly, we sequenced the normal DNA library created by ***DEEPER-Seq*** method and analyzed the data using a standard data analysis pipeline, where we directly trimmed off the single-stranded barcodes, and 78,721 SNVs were detected from the exonic sequences of normal DNA sample at a read count of 30 million (error frequency 2.6 × 10^−3^, Fig. 5A). The total number of SNVs detected from 30 million reads of the normal tissue DNA is significantly higher than what was reported on other platforms^16^. Next, we investigated if there is any bias in SNVs identified by ***DEEPER-Seq*** using the standard NGS data analysis workflow. Transition-transversion (ts/tv) ratio is routinely used to evaluate the specificity of new SNP calls. We calculated the ts/tv ratio on the target regions of ***DEEPER-Capture*** WES to be 2.766, higher than the reported ts/tv ratios of 2.0-2.1 for WGS data^33^. We then determined the ts/tv ratio in CCDS exonic regions as 3.225, which falls into the range of 3.0~3.3 for reported exonic variations^34^. The reason for ***DEEPER-Seq*** based whole exome sequencing to have a higher ts/tv ratio than reported WGS studies is because target regions of ***DEEPER-Capture*** are enriched for exons, and only contain UTRs and short flanking sequences within introns (Fig. 2A).

We then examined the accuracy of mutations identified by ***DEEPER-Seq***. Following the 4-step data analysis procedure introduced in Materials and Methods, super reads were generated after Step 3). Steps 1~3 helped to reduce the mutation frequency by over 2 orders of magnitude from 2.6 × 10^−3^ down to 2.5 × 10^−5^ by removing most PCR related errors (Fig. 5B). This result indicates that PCR related artificial mutations dramatically reduce NGS sequencing accuracy. To detect rare mutations, or even ultra-rare mutations using NGS, a correction for PCR errors is mandatory. As outlined in Step 4), we then tried to further reduce artificial errors of mutation calling by using the redundant sequence information offered by complementary DNA strands that were originally from the same DNA duplex molecule. Our results indicated that such procedure resulted in a single base mutation frequency of 1.6 × 10^−6^ (Fig. 5B). For any single base in the DNA sequences, the possibility of having exactly the same artificial error on a paired position is 1/3 × (2.5 × 10^−5^)^2^ = 2.08 × 10^−10^, which is equivalent to one artificial error per 4.8 × 10^9^ nucleotides. This is the theoretical error rate for ***DEEPER-Seq***. The total amount of DNA sequence data and the remaining amount of data after each step can be found in Table 1, where a stepwise drop of data amount is correlated to the increase of mutation calling stringency.

To determine the accuracy of variant detection by ***DEEPER-Seq*** for clinically relevant mutations, we analyzed side-by-side the WES data generated from the normal and tumor tissue pair. For all assessed heterozygous exonic positions, we filtered the result through our 4-step procedure. The filtered result showed that ***DEEPER-Seq*** identified 97 sequence variants that were exclusively detected in tumor tissue DNA sample with ≥100X coverage at different fractions. 40 moderate- to high-abundance (>5%) variants were subject to Sanger sequencing validation, and 38 were confirmed (Supplementary Table S2). Two variants failed to be validated where both allelic fractions were low and beyond the detection limit of Sanger sequencing. 57 sequence variants (with mutant allele fractions <5%) were not subject to Sanger sequencing validation at all, due to the limited sensitivity of Sanger sequencing^35^.

### Detection of ultra-rare mutations by *DEEPER-Seq* WES

SNVs can be detected with confidence only when the sequencing system’s error rate is significantly lower than the frequency of identified SNVs. Therefore, baseline error rate of an NGS pipeline is critical for its performance of detecting ultra-rare SNVs. To further assess the baseline mutation frequency of our method, we created an updated normal exome reference database for the patient based on the procedures introduced in *Supplementary Methods*. With the updated reference exome, we calculated the error rate for ***DEEPER-Seq*** method as 2.25 × 10^−10^. This error rate is very close to the theoretical error frequency of 2.08 × 10^−10^ and the method is sufficiently accurate for us to identify most ultra-rare mutations.

**Figure 6:**
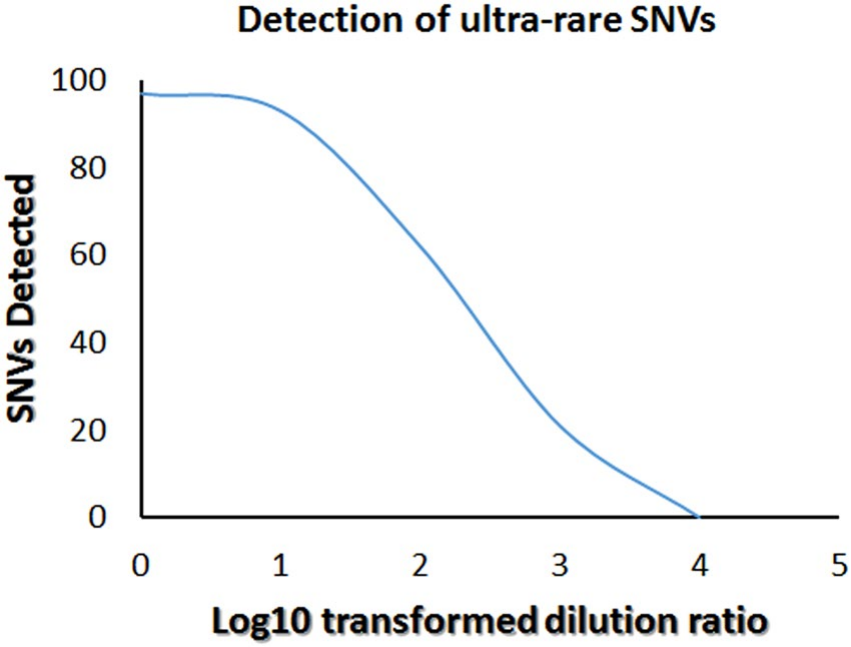
Detection of ultra-rare SNVs in libraries created from normal DNA spiked with sequentially diluted tumor DNA samples. Reduced amounts of variants were re-detected from sequentially diluted samples. No variant was re-detected from 1:10,000 diluted group. Coverage of re-sequencing is ~5,000 X.

The ultra-rare mutation detection performance of our method was then evaluated by the success rate of re-detecting the 38 validated sequence variants in the libraries created from normal DNA samples which were spiked with sequential dilutions of tumor DNA. As the dilution folds increased, as expected, we detected less and less variants (Fig. 6), and when the tumor DNA sample was diluted 1,000 folds (the diluted sample containing 0.1 ng tumor DNA and 100 ng normal DNA), we can only detect 21 out of the 38 validated variants (Supplementary Table S2). The allelic fractions of these 21 SNVs in the 1:1000 diluted sample range from 0.03% to 0.005% with an average of 0.013% (Supplementary Table S2). We did not detect any sequence variants in 1:10,000 diluted sample which may presumably due to the limitation of sequencing depth we achieved. For each sample, we performed the targeted sequencing with an average depth of 5,000×, which theoretically only allows us to see SNVs down to the frequency of 1/5000 (0.02%). To observe ultra-rare SNVs at even lower frequencies, a greater than 5000× coverage is needed. It is also helpful to design ***DEEPER-Capture*** probes targeting only a small number of genes. With a smaller number of sequencing targets, a standard ***DEEPER-Seq*** can achieve a much greater sequencing depth with a significantly improved accuracy of ultra-rare SNV calling. The extremely low baseline error rate of ***DEEPER-Seq*** allows ultra-rare SNV calling at the whole exome level with high accuracy, and the depth of NGS sequencing becomes the only limiting factor for such applications.

## Discussion

The ***DEEPER-Seq*** methodology combines the error-correcting capabilities of barcoded sequencing with an ultra-sensitive single-stranded DNA library construction and a novel highly efficient target sequence enrichment system. The ***DEEPER-Library*** can produce high quality barcoded single-stranded DNA library from very limited (<20 pg) DNA input, which allows a broader range of applications with extreme sequencing accuracy. The ***DEEPER-Capture*** highly efficiently captures target sequences from DNA library by enriching both DNA strands of target sequences simultaneously through complementary RNA probes. Efficiency of library construction and error-correction and the reasons for high enrichment performance observed from the method are discussed in Supplementary Discussion.

### Comparison to other ultra-rare SNV calling methods

At an average of 200× read depth, the ***DEEPER-Seq*** sequencing system detected 97 mutations in exonic regions, and many of which (38/40 = 95%) were then subsequently validated by Sanger sequencing (Supplementary Table S2). Sanger sequencing has a reported SNV detection sensitivity at 15% mutant allele fraction and the ones failed to be validated may be true variants but beyond the capability of Sanger^35^.

There are several technologies that can detect targeted SNVs at very low allelic fractions. CAPP-Seq could detect stage I non–small-cell lung cancer (NSCLC), with 96% specificity for mutant allele fractions down to ~0.02%^3^. Therascreen® RGQ PCR kit by Qiagen adopted an allele-specific amplification method for the detection of KRAS and EGFR mutations with almost 100% sensitivity and specificity^7^. Narayan A., *et al*. reported an approach to measure hotspot mutations in tumor DNA at the sensitivity of 1 variant in 5,000 molecules in blood using barcoded PCR based error-suppressed multiplexed deep sequencing^6^. Kinde I, *et al*. developed a Safe-Sequencing System (Safe-SeqS) that could detect and quantify rare SNVs with mutant allele fraction of 0.1%^8^. Gregory M., *et al*. reported the CypherSeq technique, which can detect rare mutations (2.4 × 10^−7^ per base pair) within specific target genes via rolling cycle amplification-based enrichment^10^. Most of these methods are based on amplification of a short and specific region of the genome to reach an extremely high read depth. Therefore, such methods are inherently limited to detect rare mutations within a few small subgenomic regions or several mutation hotspots of disease-causing genes^3, 6–8^.

For a whole exome or any targeted sequencing study, the enrichment efficiency of target regions is a critical value that determines the broadness and depth of coverage in the NGS result. In the case of low enrichment efficiency, NGS results may contain predominately PCR duplicates with high levels of artificial errors, and after error-correcting steps, the number of consolidated super reads will be extremely low, thus being not suitable for further analysis. Until now, there is no other NGS target enrichment method that could offer a comparable efficiency to what ***DEEPER-Capture*** is offering.

Taken together, it has been a dilemma that a higher depth sequencing, targeting a smaller genomic region, will result in higher sensitivity in mutation calling within limited regions; a lower depth sequencing, targeting a greater genomic region, will be much less sensitive in mutation calling, but can sequence broader regions^16^. So far, ***DEEPER-Seq*** is the only method that can identify novel ultra-rare SNVs with high sensitivity and accuracy on a whole exome sequencing scale from very limited DNA input materials (e.g. 20 pg).

### Applications of *DEEPER-Seq*

We reported ***DEEPER-Seq*** as an improved pipeline to perform NGS, particularly targeted NGS. We have demonstrated such improved performance in a human genome WES study. Aside from WES, another very important application of ***DEEPER-Seq*** would be the targeted re-sequencing of a gene panel. Targeted re-sequencing is one of the most popular NGS applications and it allows people to sequence a small cohort of gene targets to extreme depths, usually thousands of folds of coverage. And such sequencing depth can facilitate the detection of ultra-rare mutations with great sensitivity. In an ***DEEPER-Seq*** based WES study, we tried to capture the entire exome of all human genes, where an over 98% coverage with an average depth of over 200× was achieved on a standard NGS platform. More importantly, our detection limit of rare-mutation detection on whole exome scale is as low as 0.03%. For an even smaller cohort of target genes, the depth and coverage of ***DEEPER-Seq*** can be further increased, and the performance of ultra-rare mutation detection can be subsequently improved over additional several orders of magnitude.

Other than identifying ultra-rare SNVs with high sensitivity and accuracy, our method can also be adopted for gene copy number variant (CNV) assays. ***DEEPER-Library*** links a unique barcode to every single-stranded DNA molecules. Such barcode information can not only be used to label the molecules and create super reads for the purpose of reducing PCR errors, but also be used as a location marker for DNA fragments. After mapping the super reads back to human genome, the barcode on each super read can be assigned to the position where the super read sequence is mapped. Therefore, a human genome can be reconstructed by unique barcodes. Copy number information can be represented by the diversity of barcodes at subgenomic loci. More importantly, in our method, unique barcodes are specific to DNA single strands. Such information can allow further normalization of the CNV data by taking into the consideration that genomic DNA exists as duplex molecules and the density of unique barcodes for both DNA strands should match. Such calculation can massively improve the accuracy of CNV calling.

Aside from CNV analysis, large structural variants frequently observed in cancer genomes can also be analyzed in our pipeline. NGS sequencing improved by high sensitivity and deep coverage of ***DEEPER-Library*** sample preparation will provide reads covering the breakpoints with higher confidence than standard pipeline, and ***DEEPER-Capture*** RNA probes can be designed to specifically enrich subgenomic regions flanking popular genome breakpoints. A highly sensitive pipeline for translocation and large indel identification could be built based on ***DEEPER-Seq***.

In addition to applications in basic research, ***DEEPER-Seq*** has a great potential in clinical NGS fields. We have demonstrated that our method can highly efficiently construct NGS DNA libraries with very low amount of DNA materials (≤20 pg), meanwhile it can detect ultra-rare mutations with high confidence. Such features are critical for NGS based clinical diagnostics where the samples are often limited and highly heterogeneous. A typical example would be the NGS sequencing of FFPE samples. FFPE has been a standard sample preparation method for many decades. Historically archived FFPE sample is a very valuable resource for retrospective studies in biomedical research. However, due to chemical modifications during specimen preparation and chronic damages to the tissue blocks or slides over long-term storage, it has been a challenging task to conduct NGS studies with FFPE samples. Poor DNA quality and artificial sequence changes are two major issues coming along with FFPE based NGS studies. In our method, ***DEEPER-Library*** is built upon a single-stranded library protocol which was originally designed for NGS studies with fossils or damaged DNA samples^12^. Other than the feasibility of ***DEEPER-Library*** for FFPE samples, ***DEEPER-Capture*** is also offering great benefit for FFPE based WES studies. WES data have been reported to be discordant between FFPE and fresh frozen samples at lower coverage levels (~20X), however, this discrepancy can be reduced when higher coverages are achieved^36^. And recently, Allen *et al*. reported a reciprocal overlap of 90% somatic mutations between FFPE and fresh frozen tissue samples for the positions with sufficient sequencing^18^. In Allen’s study, a ***Half-DEEPER-Capture*** based capture approach was applied, where its capture efficiency is far lower than ***DEEPER-Capture*** approach as was shown in our data. With the enhanced capture efficiency by ***DEEPER-Capture***, WES studies using FFPE samples will offer comparable data quality to WES studies using fresh frozen tissues.

Our method has a great potential to discover novel low-frequency disease-causing variants in biomedical and clinical applications, and can identify more actionable therapeutic targets for patients. This method can fulfill an unprecedented level of personalized precision medicine by revealing the most complete patient genomic profile to date including high-frequency, moderate-frequency, low-frequency and particularly ultra-low-frequency mutations. Our method can also be applied in other clinical applications, like circulating DNA sequencing from body fluid samples, where only limited amount of DNA material is available. In clinical NGS applications, it is critical to construct NGS libraries from very limited amount of highly heterogeneous samples thus being less- or non-invasive; to highly efficiently enrich target sequences thereby reaching a great sequencing depth with limited cost and improved diagnostic sensitivity; and to remove artificial sequencing errors as completely as possible for the best diagnostic specificity. Our method has been demonstrated to meet these needs with great potentials in numerous NGS applications.

## Electronic supplementary material

Supplementary Information

Table S1

Table S2.
