# Supplementary material for: Targeted sequencing of both DNA strands barcoded and captured individually by RNA probes to identify genome-wide ultra-rare mutations
Source: Sci Rep. 2017 Jun 13;7:3356. doi: 10.1038/s41598-017-03448-8 (PMC5469810; doi:10.1038/s41598-017-03448-8)
Supplement: Supplementary file 1 — Supplementary Information [file 41598_2017_3448_MOESM1_ESM.pdf]

Targeted sequencing of both DNA strands barcoded and captured individually by RNA probes to identify genome-wide ultra-rare mutations

Qing Wang[1,2,3,*], Xu Wang[2], Pheobe S. Tang[1,2], Grace M. O'leary[1,2], and Ming Zhang[1,2,3,*]

[1] MyOmicsDx Inc., 600 Washington Avenue Suite 100-W, Towson, Maryland 21204, USA

[2] MyOmicsDx (Beijing) Inc., North Yong Chang Road Building # 3-3, Economic-Technological Development Area, DaXing, Beijing 100176, P.R.China

[3] Clinical Proteomics Technologies Inc., 8101 Sandy Spring Road, Laurel, Maryland 20707, USA

[*]Correspondence and requests for materials should be addressed to Q.W. (email: qwang@myomicsdx.com) or M.Z. (email: mzhang@myomicsdx.com)

Supplementary Information

This file includes:

SUPPLEMENTARY FIGURES
Supplementary Figure S1: Read statistics.
Supplementary Figure S2: Density plots of read depths.

SUPPLEMENTARY TABLES
Supplementary Table S1: 298-gene panel real-time PCR parameters.
Supplementary Table S2: Mutation and ultra-rare mutation detection by ***DEEPER-Seq***.

SUPPLEMENTARY METHODS

SUPPLEMENTARY DISCUSSION

REFERENCE

SUPPLEMENTARY FIGURES

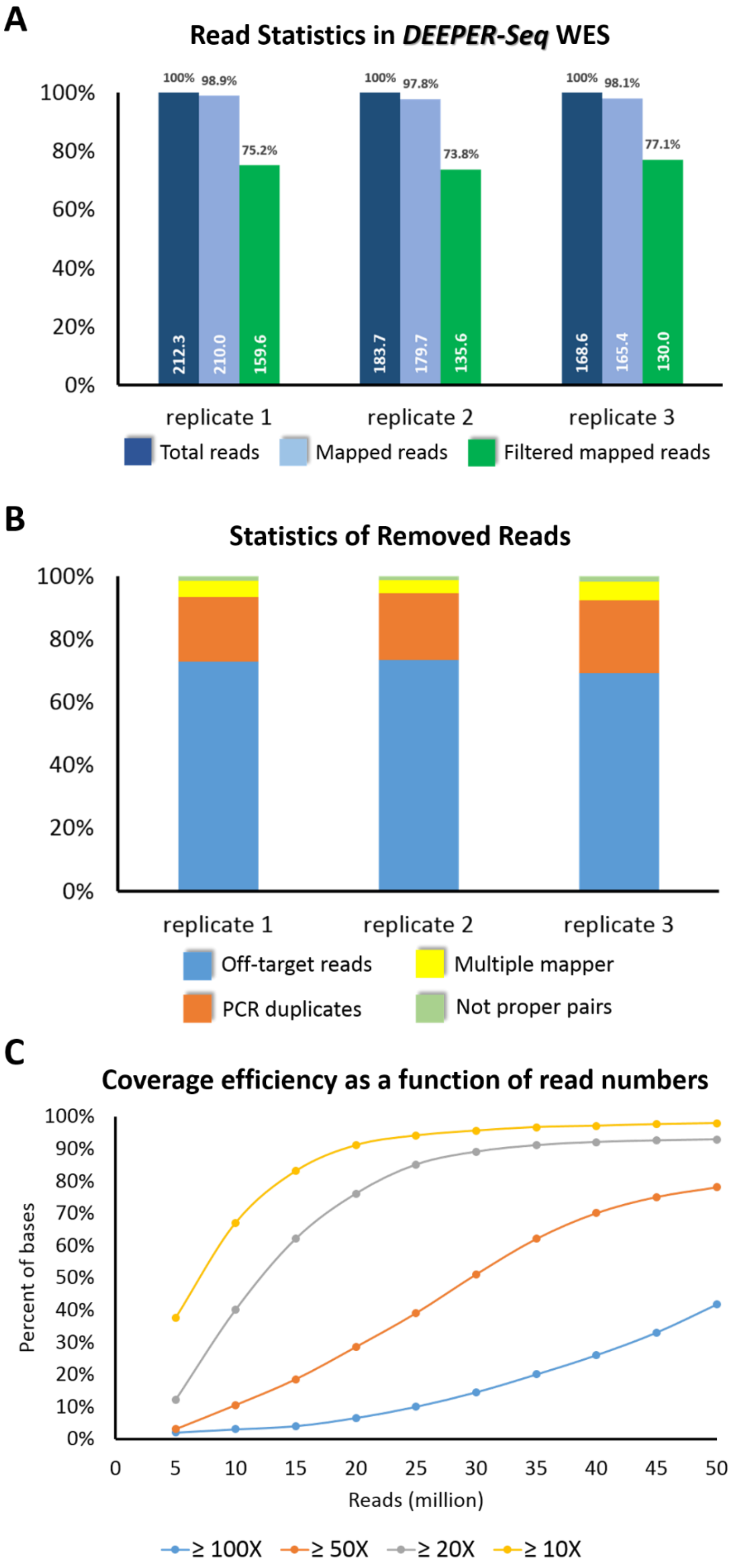

Figure S1. Read statistics. (A) Bar plot of percentage of initial reads, mapped reads and reads remained after filtering. Results were obtained from three technical replicates. Numbers of reads were shown under each bar with the unit of 1 million reads. (B) Stacked bar plot of subgroups of filtered reads in triple replicates. (C) Coverage efficiency correlation with read numbers. The percentage of target bases covered at ≥10X, ≥20X, ≥50X and ≥100X depths with 5 million to 50 million reads were shown.

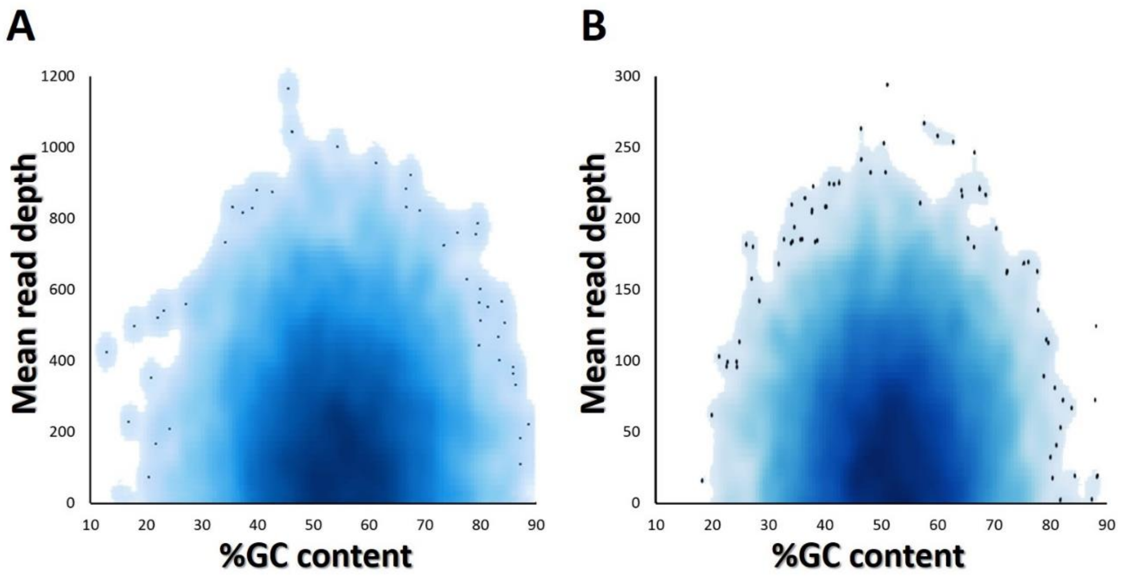

Figure S2. Density plots of read depths. Density plots were created to show GC content against normalized mean read depth for (A) ***DEEPER-Seq*** WES study with normal tissue DNA; (B) ***DEEPER-Seq*** WGS study with normal tissue DNA (without enrichment for whole exome).

SUPPLEMENTARY TABLES (attached as separate Excel documents)

Table S1. 298-gene panel real-time PCR parameters.

298 cancer related gene targets, primer pairs, amplicon sequences, amplicon GC% and amplification efficiency constant for each real-time PCR detection are listed.

Table S2. Mutation and ultra-rare mutation detection by ***DEEPER-Seq***.

Sequence variants detected by ***DEEPER-Seq***, validation results by Sanger sequencing and ultra-rare mutation re-detection results are shown and ranked by Mutant Allele Fraction.

SUPPLEMENTARY METHODS

Real-time PCR assay

Real-time PCR assays with SYBR green detection was carried out using an ABI PRISM 7500 Sequence Detection System (Applied Biosystems). Briefly, the reaction conditions consisted of 500ng of genomic DNA or DNA library products, 0.2 µM primers, and SYBR Green Real-Time PCR Master Mix (ThermoFisher Scientific) in a final volume of 20µl. Each cycle consisted of denaturation at 95°C for 15 seconds, annealing at 58.5°C for 5 seconds and extension at 72°C for 20 seconds, respectively. Gene specific primers were designed using Primer 3 [1] and their sequences are provided in Supplementary Table S1. Reactions were run in triplicate in three independent experiments. The primer pair's standard amplification curve for each gene was established through using sequential dilutions of the "+" clone constructs containing the amplicon sequence, which was originally created to generate the ***DEEPER-Capture*** RNA probes. Amplification efficiencies for 298 target amplicons were established and listed in Supplementary Table S1. Gene abundance ratios between different samples were calculated by the raising the gene specific amplification efficiency (AE) to the power of ΔC_t value between different samples. For example, the ratio (r) of gene abundance in sample A vs sample B can be calculated through real-time PCR assay by:

$$r_{(A/B)} = AE^{\Delta Ct}, \text{ where } \Delta C_t = C_{t\ (\text{sample B})} - C_{t\ (\text{sample A})}$$

Build a highly accurate reference exome for ultra-rare mutation identification

To highly accurately assess the baseline mutation frequency of ***DEEPER-Seq*** pipeline, we constructed six replicates of standard NGS DNA libraries in parallel, each using 100ng normal DNA input. We used these six replicates of exome datasets to re-build our own reference exome database for this particular patient by requesting that if the same SNV was observed in ≥ 5 out of 6 independent datasets, we considered the SNVs as germline variants and updated our reference exome sequence database. For a standard NGS pipeline, the error rate is 1%, and the chance to see exactly the same random error at a fixed position for 5 times is $(⅓ * 1\%)^5 = 4.12X10^{-13}$. This number means that if we use this approach to sequence the whole human genome once, we are presumably going to have only one artificial error, because $3 X 10^{12}$ human genome bases X $(4.12 X 10^{-13}) = 1.24$. However, we are enriching and sequencing the human exome, which is occupying only 1.5% of human genome, therefore the chance to see a single artificial error within the entire human exome is only 1.86% (=1.5%X1.24). An updated highly accurate normal exome reference database of the patient was built accordingly.

SUPPLEMENTARY DISCUSSION

DEEPER-Library offers the ultimate ability to detect ultra-rare mutation in limited amount of samples or damaged samples

The ***DEEPER-Library*** creates a large number of barcoded DNA read families (URFs), where each family arises from a single-stranded DNA molecule. After sequencing the library, DNA molecules within the URF can be identified and grouped based on the fact that they all share an identical barcode sequence (Figure 1). Only the URF with at least 3 reads and with 95% molecule members sharing the same sequence at any giving position is adopted as a read family to

generate the consensus sequence (a super read). This step efficiently removes artificial PCR errors that occur during repeated rounds of library amplification.

If an artificial error occurs at the very first step of PCR amplification, it will propagate to at most 50% of the PCR products of that sequence. Artificial variants that arise due to PCR errors or sequencing errors can be removed based on the fact that errors occur along with multiple rounds of PCR amplifications, thus being observed from only a subgroup of the reads sharing the same unique barcode. A filter can be adopted to abandon the URF whose sequence uniformity is lower than a threshold, and for this study such threshold was set as 95%. A higher threshold can further improve the sequencing accuracy, but will lead to a lower number of super reads. With a large number of high fidelity super reads collected, each super read, bearing a unique barcode, is aligned to its complementary super read by virtual of sharing a complementary consensus sequence but being differently barcoded. By mapping the super reads arising from both DNA strands individually, artificial errors in super reads can be removed in such a way that a sequence variant at a position is considered real only if a matched sequence variant can be observed at the same position from the other complementary DNA strand super read with a different barcode. The possibility for any artificial sequence variants to have a matched artificial variant at the same position from a complementary DNA strand is $< 6.45 \times 10^{-14}$ per base.

DEEPER-Capture based DNA capture enables ultimately the best capture efficiency

In 1960, DNA-RNA hybridization was reported for the first time before the term was invented [2]. Since then, numerous studies have reported that an RNA probe can bind to its complementary DNA target sequence with a much stronger affinity than a DNA probe [3,4]. In ***DEEPER-Capture***, capture efficiency is greatly improved by using a large amount of RNA probes to capture both DNA strands of the same DNA duplex molecule, simultaneously (Figure 4A). ***DEEPER-Capture*** achieves an unprecedented 29.2% capture ratio on average, and this phenomenal high efficiency is achieved presumably due to two reasons:

1) The large number of single-stranded RNA probes used in _DEEPER-Capture_ may improve the hybridization reaction. The excessive amount of RNA probes will push the balance of the binding reaction towards forming RNA-DNA duplex, and RNA duplex that can then be easily removed by RNase treatment if needed. The logistics of standard single-stranded RNA probe based capture (***Half-DEEPER-Capture***) and ***DEEPER-Capture*** can be illustrated by the following equations with the assumption that $\text{RNA-Probe}_{(-)}$ and $\text{RNA-Probe}_{(+)}$ have the complementary sequences and can capture $DNA_{(+)}$ and $DNA_{(-)}$ strands, individually:

Standard single-stranded RNA probe based capture (***Half-DEEPER-Capture***):

$DNA_{(+)}\ \mathbf{:}\ DNA_{(-)} + \text{RNA-Probe}_{(-)} \rightleftharpoons DNA_{(+)} + DNA_{(-)} + \text{RNA-Probe}_{(-)} \rightleftharpoons DNA_{(+)} : \text{RNA-Probe}_{(-)} + DNA_{(-)}$ (*)

$Rate_{\text{capturing DNA } \mathbf{single} \text{ strand}} = \boldsymbol{k_1} \cdot [DNA_{(+)}\ \mathbf{:}\ DNA_{(-)}] \cdot [\text{RNA-Probe}_{(-)}]$ (Equation 2)

*the reaction equation shows the balance of single-stranded $RNA_{(-)}$ probe capturing the single-stranded $DNA_{(+)}$ target, and the same balance holds for single-stranded $RNA_{(+)}$ probe capturing the single-stranded $DNA_{(-)}$ target (not shown here). For any given DNA sequence, only one strand, either $DNA_{(+)}$ or $DNA_{(-)}$, can be captured, not both.

Double-stranded probe capturing (***DEEPER-Capture***):

$$DNA_{(+)} : DNA_{(-)} + RNA\text{-}Probe_{(+)} + RNA\text{-}Probe_{(-)} \rightleftharpoons DNA_{(+)} + DNA_{(-)} + RNA\text{-}Probe_{(+)} + RNA\text{-}Probe_{(-)} \rightleftharpoons DNA_{(+)} : RNA\text{-}Probe_{(-)} + DNA_{(-)} : RNA\text{-}Probe_{(+)} + RNA\text{-}Probe_{(+)} : RNA\text{-}Probe_{(-)}$$

$$Rate_{capturing\ DNA\ \mathbf{double}\ strands} = \boldsymbol{k_2} \cdot [DNA_{(+)} : DNA_{(-)}] \cdot [RNA\text{-}Probe_{(+)}] \cdot [RNA\text{-}Probe_{(-)}] \quad \text{(Equation 3)}$$

As shown in the equations (2) and (3), in ***DEEPER-Capture***, the concentrations of RNA-Probe$_{(+)}$ and RNA-Probe$_{(-)}$ are adjusted to be significantly excessive as opposed to the concentration of the target DNA duplex molecules [DNA$_{(+)}$ **:** DNA$_{(-)}$]. As shown in Equation 2, the rate of the hybridization reaction between single-stranded RNA probes and single-stranded DNA molecules can be improved by increasing the [RNA-Probe$_{(-)}$] concentration. Furthermore, as shown in Equation 3, when RNA-Probe$_{(+)}$ is added, the hybridization is even more efficient by the fact that the rate is multiplied by the factor of another large concentration value [RNA-Probe$_{(+)}$].

2) *DEEPER-Capture* may improve the hybridization reaction by depleting one DNA strand, thus helping to expose the other DNA strand to a large amount of complementary RNA probes, both of which may synergistically increase the reaction constant $\boldsymbol{k_2}$ to be significantly larger than $\boldsymbol{k_1}$. When a DNA duplex is placed in a heated environment around its T_m, the two complementary DNA strands are either separated (for strands or regions with low GC content) or loosely associated (high GC regions). When one of the two complementary DNA strands is captured by an RNA probe, the other DNA strand can be more accessible to its complementary RNA probes. Therefore, ***DEEPER-Capture*** may improve target capture efficiency by achieving a much larger $\boldsymbol{k_2}$ over $\boldsymbol{k_1}$.

It has been reported that in NGS capture methods, overlapping baits improves sensitivity and are superior to an immediately adjacent or spaced design, and relatively long baits and RNA-based baits can increase capturing efficiency [5]. The ***DEEPER-Capture*** method we reported here utilizes randomly sheared massive amounts of RNA probes with their length ranging from 100 to 150nt, which are heavily overlapped and covering the target DNA regions thousands of times. We demonstrated the superior capturing efficiency of the RNA probes designed and synthesized by our pipeline. Our findings once again supported the previous observations of RNA probes in capture operation. More importantly, we reported for the first time that when the overlapping RNA probes are in excessive amount (compared to DNA molecules) and are targeting both DNA strands simultaneously, a significantly improved capture efficiency can be achieved.

Off-target enrichment was one of the biggest concerns in ***DEEPER-Capture***. The highly efficient ***DEEPER-Capture*** approach relies on a large amount of RNA probes that are overlapping with each other and are complementary to both DNA strands of the same targeted genomic region. A major side reaction would be the formation of RNA duplex molecules, RNA$_{(+)}$ **:** RNA$_{(-)}$, from two complementary RNA single strands. However, this side interaction may have only limited negative impact on the formation of DNA$_{(+/-)}$ **:** RNA-Probe$_{(-/+)}$ hybrids. Furthermore, RNA duplex molecules as well as the excessive amount of RNA probes can be removed by RNase treatment if necessary, and the captured target DNA sequences won't be affected. A major concern for off-target enrichment in NGS is the proportion of unwanted genomic DNA fragments that are being enriched through unspecific hybridization. To address this issue, we optimized capturing conditions with different buffer systems, capture reaction temperatures, incubation times and blocking primer sequences and concentrations, etc. Under the optimized condition, ***DEEPER-Capture*** showed that only an average of 16.4% of the total reads are off-target reads in a WES study (Supplementary Figure S1B). This is an acceptable ratio for most NGS applications and is

lower than other target enrichment methods [5-14]. Further improvements can be achieved with additional optimized conditions or procedures.

There are several widely used commercial kits designed to capture DNA subgenomic regions. Agilent, NimbleGen and Illumina are three major vendors in this field. Based on chemical natures of their probes, these commercially available approaches can be classified into two categories: 1) RNA probes: Agilent's SureSelect; 2) DNA probes: Roche's NimbleGen SeqCap, Illumina's TruSeq and Nextera. Several studies have been conducted to compare these capture methods in terms of their performance in WES [5,6,8,15-17]. All the platforms mentioned above can capture over 90% of the unique sequences in a WES study with a minimal sample input ranging from 50ng (Illumina Nextera) to 1.1ug (NimbleGene). Agilent SureSelect offers the only RNA probe-based (single-stranded RNA probes) capture method on the market, and has been reported to perform successful capture with down to 6.25ng input DNA to achieve ~300X mean depth of coverage with an SNV detection sensitivity >96% for high prevalence SNVs (allelic fractions >15%) [7]. As we introduced above, RNA baits have unprecedented advantages over DNA baits, such that it bindd to target DNA much stronger than DNA probes, and that RNA baits do not interfere with downstream PCR reactions and can be easily removed. Like us, Agilent adopted RNA probes, but their RNA baits are targeting only one strand of the DNA targets with very limited probe amount. However, in ***DEEPER-Capture*** we are capturing both DNA strands simultaneously with an excessive amount of probes, thus achieving an over 3 folds improved efficiency comparing to a single-stranded capture approach.

REFERENCE

1 Untergasser, A. *et al.* Primer3--new capabilities and interfaces. *Nucleic Acids Res* **40**, e115, doi:10.1093/nar/gks596 (2012).

2 Rich, A. A Hybrid Helix Containing Both Deoxyribose and Ribose Polynucleotides and Its Relation to the Transfer of Information between the Nucleic Acids. *Proc Natl Acad Sci U S A* **46**, 1044-1053 (1960).

3 Lesnik, E. A. & Freier, S. M. Relative thermodynamic stability of DNA, RNA, and DNA:RNA hybrid duplexes: relationship with base composition and structure. *Biochemistry* **34**, 10807-10815 (1995).

4 Gyi, J. I., Lane, A. N., Conn, G. L. & Brown, T. The orientation and dynamics of the C2'-OH and hydration of RNA and DNA.RNA hybrids. *Nucleic Acids Res* **26**, 3104-3110 (1998).

5 Clark, M. J. *et al.* Performance comparison of exome DNA sequencing technologies. *Nat Biotechnol* **29**, 908-914, doi:10.1038/nbt.1975 (2011).

6 Chilamakuri, C. S. *et al.* Performance comparison of four exome capture systems for deep sequencing. *BMC Genomics* **15**, 449, doi:10.1186/1471-2164-15-449 (2014).

7 Chung, J. *et al.* The minimal amount of starting DNA for Agilent's hybrid capture-based targeted massively parallel sequencing. *Sci Rep* **6**, 26732, doi:10.1038/srep26732 (2016).

8 Sulonen, A. M. *et al.* Comparison of solution-based exome capture methods for next generation sequencing. *Genome Biol* **12**, R94, doi:10.1186/gb-2011-12-9-r94 (2011).

9 Van Allen, E. M. *et al.* Whole-exome sequencing and clinical interpretation of formalin-fixed, paraffin-embedded tumor samples to guide precision cancer medicine. *Nat Med* **20**, 682-688, doi:10.1038/nm.3559 (2014).

10 Okou, D. T. *et al.* Microarray-based genomic selection for high-throughput resequencing. *Nat Methods* **4**, 907-909, doi:10.1038/nmeth1109 (2007).

11 Hodges, E. *et al.* Hybrid selection of discrete genomic intervals on custom-designed microarrays for massively parallel sequencing. *Nat Protoc* **4**, 960-974, doi:10.1038/nprot.2009.68 (2009).
12 Hodges, E. *et al.* Genome-wide in situ exon capture for selective resequencing. *Nat Genet* **39**, 1522-1527, doi:10.1038/ng.2007.42 (2007).
13 Ng, S. B. *et al.* Exome sequencing identifies the cause of a mendelian disorder. *Nat Genet* **42**, 30-35, doi:10.1038/ng.499 (2010).
14 Ng, S. B. *et al.* Targeted capture and massively parallel sequencing of 12 human exomes. *Nature* **461**, 272-276, doi:10.1038/nature08250 (2009).
15 Asan *et al.* Comprehensive comparison of three commercial human whole-exome capture platforms. *Genome Biol* **12**, R95, doi:10.1186/gb-2011-12-9-r95 (2011).
16 Meienberg, J. *et al.* New insights into the performance of human whole-exome capture platforms. *Nucleic Acids Res* **43**, e76, doi:10.1093/nar/gkv216 (2015).
17 Parla, J. S. *et al.* A comparative analysis of exome capture. *Genome Biol* **12**, R97, doi:10.1186/gb-2011-12-9-r97 (2011).
